# Economic Burden and Cost Differences Between Branded and Generic Topical Antifungals in India

**DOI:** 10.7759/cureus.111392

**Published:** 2026-06-23

**Authors:** Prateek DT, Kavitha Dongerkery, Pradnya Deolekar, Atharva Dahibhate, SriramBabu V, Safaa Mohammed Shahid, Arjun Ananthanarayan, Nidhi H Vadhavekar

**Affiliations:** 1 Pharmacology, DY Patil University, School of Medicine, Navi Mumbai, IND; 2 Dermatology, DY Patil University, School of Medicine, Navi Mumbai, IND; 3 Medicine, DY Patil University, School of Medicine, Navi Mumbai, IND

**Keywords:** cost variation, generic topical antifungals, pharmacoeconomics, pharmaco-economics, topical antifungals

## Abstract

Introduction: India's pharmaceutical market is characterized by a significant presence of both generic and branded drugs, particularly for common ailments such as superficial mycoses (SFMs), which pose a substantial public health and economic burden. Patients in tropical countries like India, which have common superficial mycoses, need prolonged treatment because of their recurrence rates after initial treatment and due to incomplete therapy. Given the high variability in drug pricing and the imperative for cost-effective healthcare, a pharmacoeconomic analysis comparing generic and branded topical antifungals in India is crucial to inform prescribing practices and optimize resource allocation.

Methods: The price comparison of various topical antifungal medications from different brands was conducted by using the latest information from the "Monthly Index of Medical Specialties" August to October 2025, and 1mg online pharmacy and Jan Aushadhi website. The study calculated the total expenses for 30 mL and 30 g dosage forms, which included cream, ointment, lotion, eye drops, and shampoo products of each drug brand. We conducted a comparison between different drug brands through their cost ratio, and percentage cost variation (PCV) analysis was done, keeping generic medicines prices (retrieved from the Jan Aushadhi website) as baseline values.

Results: The data analysis showed a significant variation in the costs of different brands of topical antifungals in the Indian market. After analysis, we identified itraconazole 1% ointment to have the highest cost variation at 8335.1%, followed by clotrimazole dusting powder (4740%). Ketoconazole 2% cream, bifonazole 1% lotion, and sertaconazole shampoo showed the smallest variation at 3.29%, 3.5%, and 11.3%, respectively. When generic and branded topical antifungals were compared, the highest cost variation was seen for clotrimazole 1% cream (3431.7%), and the least variation was observed for ketoconazole 2% powder (135.4%). When combination topical antifungals were compared, the highest percentage cost variation of 1479.4% was seen for clotrimazole 1%w/w and beclomethasone dipropionate 0.025%w/w cream, and the least percentage cost variation of 38.8% was seen for terbinafine (1% w/w) + ciprofloxacin (1% w/w) + metronidazole (1% w/w) + clobetasol (0.05% w/w).

Conclusion: The market for topical antifungal agents demonstrates substantial price variation among available products. Regulatory authorities, pharmaceutical manufacturers, and clinicians must collaborate to achieve optimal reductions in drug costs. Strict implementation of cost regulation policies, along with increased awareness among clinicians regarding the rational selection of cost-effective therapies, is essential.

## Introduction

Drug therapy is an essential component of medical care. In India, reports of superficial and systemic fungal infections are rising as a result of environmental, behavioral, and treatment-related factors [1]. Superficial mycoses (SFMs) are the most prevalent type of infection globally, affecting an estimated 20%-25% of the world's population. These infections are typically anthropophilic *Trichophyton* infections [2]. In a country like ours, where heat and humidity are high for the majority of the year, it is more common. SFMs are transmitted through three different methods, which include direct human contact and contact with infected soil and infected animals [3]. In recent years, antifungal therapy has experienced a significant transformation. Current therapeutic agents can be broadly divided into two categories: synthetic medications such as azoles and fluorinated pyrimidines, and naturally occurring antifungals and echinocandins [4]. Fungal infections are becoming more common due to immunosuppression brought on by cancer chemotherapy, organ transplants, acquired immunodeficiency syndrome (AIDS), excessive corticosteroid use, and antibiotic misuse. SFMs are significant due to their global distribution, epidemiology, and morbidity, and require long-term therapy, which increases the cost of therapy [5], whereas systemic fungal infections are potentially fatal [6]. India's total population of approximately 1.4 billion people results in daily consumption of 1.26 million defined daily doses (DDD), which equals 460 million DDDs annually. This increase in antifungal medication use has resulted in higher economic costs for the country [7].

The Indian market provides various topical antifungal medications, which are used to treat superficial fungal infections (SFIs), with clotrimazole, ketoconazole, miconazole, luliconazole, eberconazole, sertaconazole, oxiconazole, fenticonazole, terbinafine, and amorolfine available in different dosage forms [8]. The available systemic antifungal medications include fluconazole, itraconazole, ketoconazole, terbinafine, and griseofulvin, which come in various topical and oral dosage forms [9].

India has one of the largest pharmaceutical markets in the world, with a broad range of branded and generic antifungal medications available for clinical use [10]. The presence of multiple brands and formulations often makes the selection of an appropriate and affordable treatment challenging for clinicians. Considering these factors, the present study was conducted to evaluate and compare the prices of different brands and dosage forms of topical antifungal agents available in the Indian market.

## Materials and methods

Methodology

The present study was a descriptive, cross-sectional pharmacoeconomic analysis conducted over a time period of three months (August-October 2025) to study cost variations among branded and generic topical antifungal formulations that are available in the Indian market.

Data sources and collection

The Current Index of Medical Specialities (CIMS), June 2025 edition, the 1mg online pharmacy platform, and the Pradhan Mantri Bhartiya Janaushadhi Pariyojana (PMBJP) website were among the publicly available sources from which information on drug costs and availability was gathered. We used the 1mg platform as our data source because it is one of India's biggest digital healthcare marketplaces, and it gives readily reachable and current information about drug prices, brand names, formulations, pack sizes, and manufacturers. With this, we can make a more standardized but also real-life comparison of the costs for topical antifungal medications across many marketed products; additional sources were used for cross-verification.

Topical antifungal medications that are frequently prescribed for superficial fungal infections were included. Fixed-dose combinations (FDCs) and single-drug formulations with the same dosage and strength were taken into consideration. Only preparations with a standard pack size of 30 g or 30 mL were included for consistent comparison. Drugs with inconsistent pack sizes or missing pricing information were not included. The number of brands available, the lowest and maximum prices, and the corresponding generic prices (if available) were noted for each formulation. Microsoft Excel 2021 (Microsoft Corp., Redmond, WA) was used to systematically tabulate the data.

Outcome measures

Cost ratio and percentage cost variation (PCV) were the main outcome measures. The ratio of the highest to the lowest price for the same drug formulation was used to compute the cost ratio. The following formula was used to determine the percentage cost variation.

The percentage cost variation among different brands of the same drug was calculated to assess the extent of price disparity. The maximum and minimum costs for each drug formulation were identified from available pricing sources. The percentage cost variation (PCV) was computed using the following formula:

\begin{equation} \text{PCV} = \left( \frac{C_{\max} - C_{\min}}{C_{\min}} \right) \times 100 \end{equation}

where "C_{\max}" represents the maximum cost and "C_{\min}" represents the minimum cost of a particular drug formulation. This method allows for quantification of the relative difference in pricing and facilitates comparison across various drug categories.

Prices for combination antifungal formulations and branded and generic medications were also compared.

Statistical analysis

Data were summarized using descriptive statistics, and the findings were presented as ranges, percentages, and frequencies. The relationship between the chosen variables (e.g., minimum/generic cost as the independent variable and maximum/branded cost as the dependent variable) was evaluated using linear regression analysis. So, we have used analysis of variance (ANOVA) to check the mean costs across several groups of topical antifungal formulations. The idea was just to compare how the average costs looked in each group.

The StatsKingdom website was used to calculate linear regression analysis and a 95% predicted interval. Linear regression analysis was performed to evaluate trends over time. The regression equation was expressed as follows:

\begin{equation} y = a + bX \end{equation}

where "b" represents the slope of the regression line, estimated using the least squares method, and "a" denotes the intercept. The slope indicates the magnitude and direction of change in the dependent variable (y) for a unit change in the independent variable (x).

The regression model's goodness-of-fit assessment used the coefficient of determination, R², while the correlation coefficient R assessment determined the strength of the relationship between variables. The model's statistical significance was assessed through analysis of variance (ANOVA) testing, which calculated both F-statistic and p-value results; any p-value result below 0.05 established statistical significance.

A 95% prediction interval was calculated to determine the range of values that individual observations would typically exhibit. The standard error of estimate and t-distribution at 95% confidence level were used to derive these results. The system produced graphical displays of the regression line together with its prediction intervals.

Ethical considerations

The study has not included any human participants or identifiable personal data; therefore, ethical approval was not required for this study.

## Results

A total of 7,447 brands representing 18 topical antifungal agents in various formulations were identified and analysed. The minimum cost, maximum cost, cost ratio, and percentage cost variation for branded topical antifungal preparations are presented in Table 1. 

**Table 1:** Maximum and minimum cost, cost ratio, and percentage cost variation of branded topical antifungals in different forms The maximum cost represents the highest price point for branded topical antifungal products that exist in the market. The minimum cost indicates the lowest price for the same formulation. The cost ratio shows price disparity through its calculation, which divides the maximum cost by the minimum cost. Percentage cost variation shows the relative difference between maximum and minimum costs to demonstrate how different brands of the same formulation vary across different dosage forms. Tabulated using Microsoft Excel 2021

Drug	Formulations	Doses	Manufacturing companies	Minimum cost (Rs)	Maximum cost (Rs)	Cost ratio	% cost variation
Amphotericin B	Gel (30 g)	0.1% w/w	2	526	1854	3.52	252.4
	Vaginal gel	0.1% w/w	1	588	588	1	0
Ketoconazole	Soap	2% w/w	535	13.6	96.9	7.12	612.5
	Cream	2% w/w	460	48	206.3	4.29	3.29
	Shampoo	2% w/w	373	59.7	142.8	2.39	139.1
	Lotion	2% w/w	193	18.75	141.9	7.56	656.8
	Solution	2% w/w	30	19.05	157.5	8.26	725.7
	Dusting powder	2% w/w	28	37	61.8	1.67	67
	Ointment	2% w/w	27	138.4	288	2.08	108
	Gel		6	57.6	329	5.71	471.1
	Bodywash	2% w/w	4	91.5	118.8	1.29	29.8
Nystatin	Ointment	1 lakh IU	1	303.8	303.8	1	0
	Cream	100000 IU	1	27.6	144	5.21	421.7
Clotrimazole	Cream	1% w/w	797	35.4	837	23.64	2264.4
	Ear drops		246	36.3	591	16.2	1528
	Dusting powder	1% w/w	182	7.5	363	48.4	4740
	Lotion	1% w/v	121	50.92	240	4.71	371.3
	Ointment	1% w/v	105	26.8	435	16.23	1523.1
	Mouth paint	1% w/v	35	42.5	201.3	4.73	373.6
	Soap	1%w/w	23	14.8	441	29.79	2879.7
	Vaginal gel	2% w/w	17	48	155	3.22	222.9
	Eye drops		10	122.4	786	6.42	542.1
	Vaginal cream	2% w/w	12	32.5	204	6.27	527.6
	Topical solution	1%w/v	10	31	109.5	3.53	253.2
Miconazole	Cream	2% w/w	704	13.65	189	13.85	1284.61
	Ointment	2% w/w	89	27.91	59	2.11	111.34
	Gel	2% w/w	21	48.2	199	4.12	312.8
	Lotion	2%w/w	14	53.2	264	4.96	396.2
	Dusting powder	2%w/w	8	35.6	87.3	2.45	145.2
	Shampoo	2%	4	30	39.75	1.32	32.5
	Vaginal gel	2%	3	33.75	105	3.11	211.1
	Powder	2%w/w	2	58	72	1.24	24.1
	Soap		1	30	30	1	0
	Spray		1	69	69	1	0
	Mouth paint		1	94.2	94.2	1	0
Terbinafine	Cream	1% w/w	509	27.6	610	22.1	2110.1
	Ointment	1% w/w	54	93	378	4.06	306.4
	Dusting powder	1% w/w	36	30	89.7	2.99	199
	Lotion	1% w/w	28	124	150	1.2	20.9
	Gel	1% w/w	8	178	393	2.2	120.7
	Powder	1% w/w	3	74.8	88.8	1.18	18.7
	Solution	1% w/w	2	144.6	161.4	1.11	10.4
	Spray	1%w/v	2	120	284.8	2.37	137.3
Voriconazole	Powder for eye drop	1%w/v	2	2,094	2910	1.38	38.9
Itraconazole	Cream	1%w/w	292	78	460	5.89	489.7
	Dusting powder	1%w/w	51	36.3	187.2	5.15	415.7
	Gel	1%w/w	30	39.6	290	7.32	632.3
	Ointment	1%w/w	24	26.2	2210	84.3	8335.1
	Soap		22	11.92	67.2	5.63	463.7
	Powder	1%w/w	8	14.2	78	5.49	449.2
	Eye drop	1%w/w	3	465.6	756	1.62	62.3
	Ophthalmic suspension		2	864	1,608	1.86	86.1
Luliconazole	Cream	1%w/w	1296	223.2	533	2.38	138.7
	Lotion	1% w/v	238	84	970	11.54	1054.7
	Ointment	1%	54	59.5	312	5.24	424.3
	Soap		32	27.6	140.5	5.09	409
	Dusting powder	1%	25	48	359	7.47	647.9
	Gel	1% w/w	9	272	351.6	1.29	29.2
	Shampoo	1%	6	96.9	136.8	1.41	41.1
	Solution	1%w/v	1	233	233	1	0
Sertaconazole	Cream	2% w/v	152	61.8	410	6.63	563.4
	Lotion	2%w/v	14	181	374	2.06	106.6
	Dusting powder	2%w/w	4	88.8	139.8	1.57	57.4
	Shampoo	2%	4	159	177	1.11	11.3
	Ointment	2%w/w	3	240	286	1.19	19.1
	Solution	2%w/v	2	366	502	1.37	37.1
	Gel		1	450	450	1	0
	Spray	2%w/v	1	210	210	1	0
Oxiconazole	Cream	1%	24	85	288	3.38	238.8
	Lotion	1%	5	87.23	209	2.39	139.5
Butenafine	Cream	1% w/w	6	84.14	284	3.37	237.5
Tolnaftate	Cream	1% w/w	150	35.4	482	13.6	1261.5
	Ointment	1% w/w	13	82	268	3.26	226.8
	Solution	10mg/ml	3	16.8	182.4	10.8	985.7
Econazole	Cream	1%w/w	7	87	189	2.17	117.2
	Ointment	1%w/w	1	38.3	38.3	1	0
Ciclopirox	Cream	1%w/w	44	40.44	265	6.55	555.2
	Shampoo	1%	9	78	132.5	1.69	69.8
	Lotion	1%	7	191	295	1.54	54.4
	Gel	1%	1	155.2	155.2	1	0
	Ointment	1%	1	74.9	74.9	1	0
	Solution	1%w/v	1	160.2	160.2	1	0
Amorolfine	Cream	0.25%	158	67.6	476	7.04	604.1
	Nail lacquer	5%	6	306.5	708	2.3	130
	Lotion	0.25%	5	246	361	1.46	46
	Gel	0.25%	1	246	246	1	0
	Solution	0.25%	1	328.13	328.13	1	0
Bifonazole	Cream	1%	16	140	273	1.95	95
	Lotion	1%	2	126	130.5	1.03	3.5
	Shampoo	1%	1	290	290	1	0

Among the single-agent topical antifungal formulations, itraconazole 1% ointment exhibited the highest percentage cost variation (8335.1%) among 24 marketed brands, followed by clotrimazole 1% dusting powder (4740%), clotrimazole 1% cream (2264.4%), and terbinafine 1% cream (2110.1%). In contrast, the lowest percentage cost variation was observed with ketoconazole 2% cream (3.29%) and bifonazole 1% lotion (3.5%). Several formulations, including amphotericin B vaginal gel, nystatin ointment, luliconazole solution, and certain ciclopirox and amorolfine formulations, were represented by a single manufacturer and therefore demonstrated no cost variation (Table 1).

**Figure 1:**
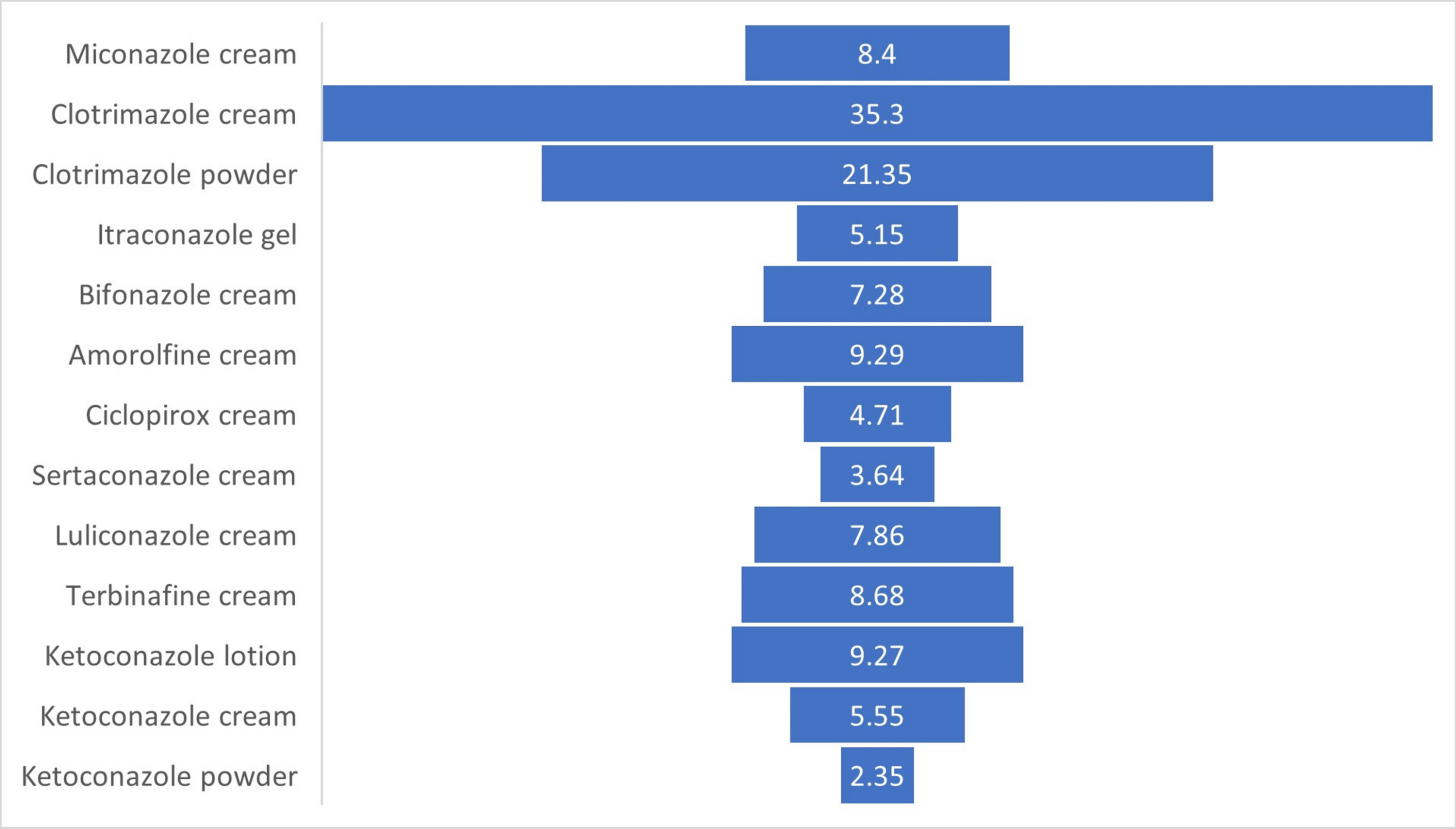
Cost variation ratios among topical antifungal preparations available in India Clotrimazole cream exhibited the highest cost ratio (35.3), indicating that the most expensive brand costs more than 35 times the least expensive brand. Similarly, clotrimazole powder showed a cost ratio of 21.35. Funnel chart made with Microsoft Excel 2021

The cost differences between branded and generic topical antifungal preparations are summarized in Table 2. Clotrimazole 1% cream demonstrated the greatest variation between generic and branded products, with a percentage cost variation of 3431.7%, followed by clotrimazole 1% powder (2035.2%). The lowest variation was observed for ketoconazole 2% powder (135.4%). The relative cost ratios of branded and generic topical antifungal preparations are illustrated in Figure 1. Clotrimazole cream showed the highest cost ratio, indicating substantial differences between generic and branded pricing.

**Table 2:** Cost variation of branded and generic topical antifungal drugs Cost variation refers to the difference in prices of the same topical antifungal drug across various branded and generic formulations available in the market. Tabulated with Microsoft Excel 2021

Drug	Strength	Generic price (A)	Minimum cost (Rs)	Maximum cost (Rs) (B)	Cost difference (B-A)	% cost variation
Miconazole cream	2%	22.5	35.6	189	166.5	740
Clotrimazole cream	1%	28.88	86.4	1020	991.1	3431.7
Clotrimazole powder	1%	17	29.2	363	346	2035.2
Itraconazole gel	1%	56.26	39.6	290	233.7	415.3
Bifonazole cream	1%	37.5	140	273	235.5	628
Amorolfine cream	0.25%	51.27	67.6	476	424.7	828.3
Ciclopirox cream	1%	56.2	35.22	265	208.8	371.5
Sertaconazole cream	2%	112.5	132	410	297.5	264.4
Luliconazole cream	1%	67.8	223.2	533	465.2	686.1
Terbinafine cream	1%	70.22	27.6	610	539.7	768.5
Ketoconazole lotion	2%	15.3	18.75	141.9	126.6	827.4
Ketoconazole cream	2%	37.14	48	206.3	169.16	455.7
Ketoconazole powder	2%	26.25	37	61.8	35.55	135.4

The pricing characteristics of combination topical antifungal preparations are shown in Table 3. Among the evaluated combinations, clotrimazole 1% w/w and beclomethasone dipropionate 0.025% w/w cream demonstrated the highest percentage cost variation (1479.4%), whereas the combination of ciprofloxacin, metronidazole, terbinafine, and clobetasol exhibited the lowest variation (38.8%). Considerable variation was also observed with the miconazole 2% w/w and fluocinolone acetonide 0.01% w/w ointments, which showed a percentage cost variation exceeding 1000% (Table 3).

**Table 3:** Maximum and minimum cost, cost ratio, and percentage cost variation of branded combination topical antifungals in different forms The maximum cost represents the highest retail price for a particular branded combination topical antifungal product, while the minimum cost shows the lowest retail price for the same product. The cost ratio uses the maximum cost and the minimum cost to calculate price differences between different brands. Tabulated with Microsoft Excel 2021

Drug	Manufacturing companies	Minimum cost (Rs)	Maximum cost (Rs)	Cost ratio	% cost variation
Ketoconazole 2% w/v and zinc pyrithione 1% w/v shampoo	7	28.11	137.5	4.89	389
Betamethasone valerate 0.12%w/w, gentamicin 0.1%w/w, and miconazole nitrate 2%w/w cream	18	30	116.8	3.89	289
Clobetasol propionate 0.05%w/w, gentamicin 0.1%w/w, and miconazole nitrate 2%w/w cream	28	25.5	171	6.7	570
Miconazole 2%w/w and fluocinolone acetonide 0.01%w/w ointment	7	46.2	532.8	11.5	1053.2
Clobetasol propionate 0.05%w/w, neomycin 0.50%w/w, miconazole 2%w/w, and chlorocresol 0.10%w/w cream	30	55.2	240	4.34	334.7
Clotrimazole 1%w/w and beclomethasone dipropionate 0.025%w/w cream	26	32.1	507	15.7	1479.4
Beclometasone (0.025% w/w), neomycin (0.5% w/w), and clotrimazole (1% w/w) cream	34	64.2	176.8	2.75	175.3
Beclometasone (0.025% w/w) and ketoconazole (2% w/w)	34	37.6	217	5.77	477.1
Ciprofloxacin (1% w/w), metronidazole (1% w/w), terbinafine (1% w/w), and clobetasol (0.05% w/w)	2	115.2	160	1.38	38.8
Terbinafine and mometasone	69	73.5	542	7.37	637.4

**Figure 2:**
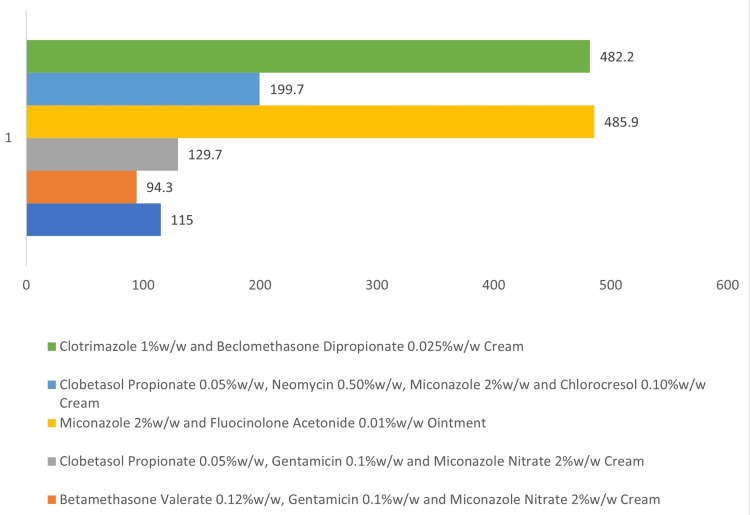
Cost variation of branded and generic topical combination antifungals Demonstrates how price differences occur between two products that share the same components and strength and therapeutic effect of the original drug. Clustered bar made with Microsoft Excel 2021

A comparison of generic and branded combination topical antifungal preparations is presented in Table 4. The highest percentage cost variation was observed for Clotrimazole 1% w/w and Beclomethasone Dipropionate 0.025% w/w cream (1947.6%), while the lowest variation was recorded for Clobetasol Propionate 0.05% w/w, Gentamicin 0.1% w/w, and Miconazole Nitrate 2% w/w cream (314.4%). The differences in pricing between generic and branded combination products are further illustrated in Figure 2.

**Table 4:** Cost variation of branded and generic topical combination antifungal drugs The study shows how different drugs with the same active ingredients, strengths, and dosage forms result in different price points, which demonstrates the economic impacts and financial gains that come from using affordable generic medications. Tabulated with Microsoft Excel 2021

Drug	Manufacturing companies	Generic cost (Rs)	Maximum cost (Rs)	Cost ratio	% cost variation
Ketoconazole 2% w/v and zinc pyrithione 1% w/v shampoo	7	22.5	137.5	6.11	511.1
Betamethasone valerate 0.12%w/w, gentamicin 0.1%w/w, and miconazole nitrate 2%w/w cream	18	22.5	116.8	5.19	419.1
Clobetasol propionate 0.05%w/w, gentamicin 0.1%w/w, and miconazole nitrate 2%w/w cream	28	41.26	171	4.14	314.4
Miconazole 2%w/w and fluocinolone acetonide 0.01%w/w ointment	7	46.88	532.8	11.36	1036.5
Clobetasol propionate 0.05%w/w, neomycin 0.50%w/w, miconazole 2%w/w, and chlorocresol 0.10%w/w cream	30	40.23	240	5.96	496.5
Clotrimazole 1%w/w and beclomethasone dipropionate 0.025%w/w cream	26	24.76	507	20.4	1947.6

**Figure 3:**
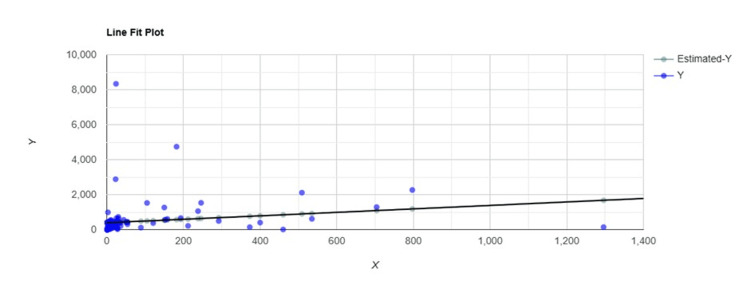
Exploratory linear regression depicting good to fitness line X axis: number of manufacturing companies, Y axis: percentage cost variation The analysis demonstrated a weak positive correlation (R = 0.183) with a low coefficient of determination (R² = 0.0336), indicating that only 3.4% of the variability in Y was explained by X [11]. Regression analysis performed using an online statistical tool (StatsKingdom)

To evaluate whether the number of manufacturing companies influenced price variation, linear regression analysis was performed using the number of manufacturers as the independent variable (X) and percentage cost variation as the dependent variable (Y). The scatterplot and fitted regression line are presented in Figure 3. A weak positive correlation was observed between the two variables (R = 0.183), with a coefficient of determination (R² = 0.034). The regression equation obtained was as follows:

Ŷ = 389.17 + 0.99X

The analysis indicated that only 3.4% of the variability in percentage cost variation could be explained by the number of manufacturing companies.

Linear regression analysis demonstrated a weak positive association between X and Y (R = 0.183, R² = 0.034). The model explained only 3.4% of the variability in the dependent variable, indicating limited explanatory power. ANOVA showed that the regression model was not statistically significant (F(1,90) = 3.13, p = 0.080) (Table 5). These findings suggest that X is not a major determinant of Y and that variability in Y is likely influenced by additional factors not captured in the model.

**Table 5:** ANOVA for linear regression model ANOVA: analysis of variance, DF: degrees of freedom, SS: sum of squares, MS: mean square, F: F-statistic Linear regression analysis demonstrated a weak positive association between X and Y (R = 0.183, R² = 0.034). This model explains only 3.4% of the variability in the dependent variable, indicating limited explanatory power. ANOVA showed that the regression model was not statistically significant (F(1,90) = 3.13, p = 0.080) [12]. ANOVA performed using an online statistical tool (StatsKingdom)

Source	DF	Sum of square	Mean square	F statistic (df_1_,df_2_)	P-value
Regression (between ŷ_i_ and ȳ)	1	3546016.1288	3546016.1288	3.1254 (1,90)	0.08047
Residual (between y_i_ and ŷ_i_)	90	102113016.2029	1134589.0689		
Total (between y_i_ and ȳ)	91	105659032.3317	1161088.2674		

**Figure 4:**
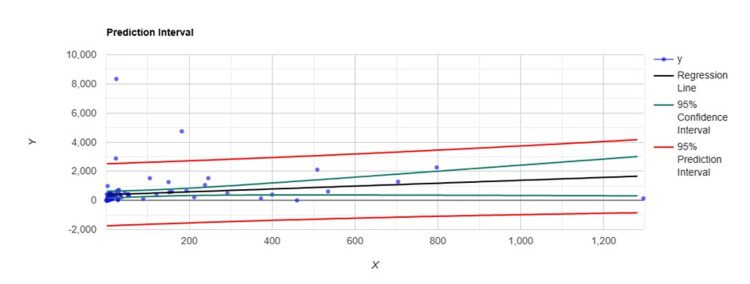
95% confidence interval and 95% prediction interval The linear regression plot demonstrates how X and Y are connected through the black regression line, which shows actual data points and 95% confidence intervals and 95% prediction intervals. The results show that there was significant variation between the two variables, and their relationship was weakly established [13]. 95% confidence interval and 95% prediction interval calculated using an online statistical tool (StatsKingdom)

The regression plot with 95% confidence and prediction intervals is shown in Figure 4 (performed using the online statistical tool StatsKingdom). The 95% confidence interval (green lines) around the regression line shows a narrow width, which demonstrates high accuracy of the estimated mean response. The 95% prediction interval (red lines) shows a much wider width, which demonstrates that individual observations will show high levels of unpredictable variation.

The data points show most observations concentrated at lower X values, which reveals that the data shows uneven distribution. The wide prediction interval and point spread demonstrate that the model has poor accuracy for predicting individual values, which matches the weak correlation and low explanatory power shown in the regression analysis.

## Discussion

In the healthcare system, evaluating prescription patterns is crucial. Drug availability and cost have an impact on prescriptions. The cost of the medication is one of the key elements that affects patient compliance [14]. Despite not being a fatal illness, SFI has a significant impact on society's financial burden. Drug resistance and a longer course of treatment to cure the infection have resulted from irrational use, incorrect dosage and duration, and the use of FDC of topical antifungal agents with steroids. This ultimately results in a very high treatment cost burden for the patient. Pharmaceutical companies attribute the high cost of their products to the high expense of research and development. However, overhead costs and product promotion cost a significant amount of money [15].

According to this study, we discovered that the majority of the commonly used topical antifungal medications have a high cost ratio. The range of cost ratios is low, from 1.03 to 84.3, indicating a significant price disparity between different brands. A similar pattern can be seen in the percentage cost variation, which ranges from a minimum of 3.29% to a maximum of 8335.1%. These results are consistent with the findings of the previous studies [16]. Whereas this was the difference between the brands, there was a huge variation between the generic and branded topical antifungals as well.

The results of this study line up with earlier pharmacoeconomic assessments of antifungal medicines in India, which have shown a rather wide price variation between brands of the same antifungal agent. Tiwari et al. noted clear differences in cost across antifungal formulations, and in fact, a number of these drugs had percentage cost swings that went beyond 100%, pointing toward the heavy financial strain tied to antifungal treatment [17]. Our study extends these observations a bit by looking more closely at topical antifungal preparations that are currently circulating in India, basically using the latest market data. We saw a strong variation across formulations such as clotrimazole, luliconazole, and terbinafine, so it implies that there are still meaningful pricing gaps even though generic products are becoming more common. The differences between what we found right now and what was reported in earlier studies could be explained by shifts in market competition, the arrival of more recent brands, changes in how clinicians prescribe, and even the periodic revisions that happen in pharmaceutical pricing [16]. Spoorthy et al. similarly reported wide cost variation among topical antifungal preparations used for superficial fungal infections and advocated stricter price regulation and greater awareness among prescribers regarding drug costs [16]. The persistence of such variation across multiple studies conducted over different time periods indicates that affordability remains a significant concern in antifungal therapy in India [18].

Recently, the Indian government opened generic pharmacies across the nation, offering medications at lower prices. To encourage prescribers, pharmacists, and consumers to use generic medications, the quality of the less expensive generic medications offered in these stores should be evaluated and contrasted with well-known brands [19].

Pharmacoeconomic analysis aids in therapeutic decision-making, formulary decision-making, program justification, drug policy decisions, and treatment guidelines, all of which ultimately benefit society by lowering healthcare costs and increasing the availability of reasonably priced medications [20].

Therefore, in order to address the issue of the enormous variation in drug costs, pharmaceutical companies, doctors, pharmacists, regulatory bodies, and the general public at large must work together.

Limitations of the study

The research contains multiple restrictions, which affect its results. First, the analysis is done by using drug price data that is obtained from open sources, yet this method fails to capture ongoing market price changes and regional price differences throughout India. Second, only standard pack sizes (30 g/30 mL) were included to ensure uniformity, which may have excluded other commonly used formulations and pack sizes.

This study assessed cost differences between groups, but it failed to evaluate essential factors, which include safety, clinical effectiveness, and patient medication adherence that guide treatment decisions. The regression analysis showed a weak relationship between variables, which lacked statistical significance; thus, it limited the model's ability to make predictions. The results of the study were affected by outliers, which created dataset variability and the dataset itself.

## Conclusions

This study demonstrated substantial price variation among marketed topical antifungal formulations in India, despite the availability of products containing identical active ingredients, strengths, and dosage forms. Such marked cost disparities can impose a significant financial burden on patients, potentially affecting treatment adherence and clinical outcomes, particularly in conditions requiring prolonged therapy. The findings underscore the need for greater price transparency, rational prescribing practices, and enhanced awareness among healthcare professionals regarding cost-effective therapeutic alternatives. Strengthening regulatory oversight and promoting the prescription of economically viable brands may contribute to reducing out-of-pocket expenditure and improving access to antifungal treatment. Overall, regular pharmacoeconomic evaluation of commonly prescribed medications is essential to support evidence-based, cost-conscious healthcare decision-making in India.

## References

[1] Bhargava S, Chakrabarty S, Damodaran RT, Saikia PK, Shenoy M, Bangale N, Shah P (2023). Rising burden of superficial fungal infections in India and the role of Clotrimazole for optimal management. Indian J Clin Exp Dermatol.

[2] James WD, Elston DM, Berger TG (2016). Andrews’ Diseases of the Skin: Clinical Dermatology.

[3] Li Q, Li J, Zhi H (2024). Epidemiological survey of 32,786 culture-positive dermatophytosis cases in Hangzhou from 2018 to 2023. Mycopathologia.

[4] Murray PR, Rosenthal KS, Pfaller MA (2013). Medical Microbiology. Medical microbiology. 7th ed. Philadelphia.

[5] DA S, Goyal R, Bhattacharya SN (2007). Laboratory-based epidemiological study of superficial fungal infections. J Dermatol.

[6] Kelly BP (2012). Superficial fungal infections. Pediatr Rev.

[7] Pathadka S, Yan VK, Neoh CF (2022). Global consumption trend of antifungal agents in humans from 2008 to 2018: data from 65 middle- and high-income countries. Drugs.

[8] Sahni K, Singh S, Dogra S (2018). Newer topical treatments in skin and nail dermatophyte infections. Indian Dermatol Online J.

[9] Sheehan DJ, Hitchcock CA, Sibley CM (1999). Current and emerging azole antifungal agents. Clin Microbiol Rev.

[10] Yashaswini P, Geetha A, Prashanth BV (2019). Analysis of pricing of oral antiviral drug formulations available in Indian market. Natl J Physiol Pharm Pharmacol.

[11] (2026). StatsKingdom: Linear regression line fit plot calculator. https://www.statskingdom.com/linear-regression-calculator.html.

[12] (2026). StatsKingdom: Linear regression and ANOVA calculator. https://www.statskingdom.com/regression-calculator.html.

[13] (2026). StatsKingdom: Linear regression prediction interval calculator. https://www.statskingdom.com/regression-calculator.html.

[14] Yusuf M, Sharma V, Pathak K (2014). Nanovesicles for transdermal delivery of felodipine: development, characterization, and pharmacokinetics. Int J Pharm Investig.

[15] Roy V, Gupta U, Agarwal AK (2012). Cost of medicines & their affordability in private pharmacies in Delhi (India). Indian J Med Res.

[16] HV S, Padma L, BP S (2021). Analysis of cost of various topical and oral antifungal drugs for superficial fungal infections available in India. Int J Basic Clin Pharmacol.

[17] Tiwari A, Reddy P, Goyal C (2016). Cost analysis of antifungal drugs available in India: a pharmacoeconomic perspective. Indian J Pharm Pharmacol.

[18] Gupta P, Sankdia RK, Marko S, Dubey L (2022). A cost variation analysis of oral and topical antifungal agents available for the treatment of superficial fungal infections in India. Asian J Pharm Clin Res.

[19] Das M, Choudhury S, Maity S, Hazra A, Pradhan T, Pal A, Roy RK (2017). Generic versus branded medicines: an observational study among patients with chronic diseases attending a public hospital outpatient department. J Nat Sci Biol Med.

[20] Ahmad A, Patel I, Parimilakrishnan S, Mohanta GP, Chung H, Chang J (2013). The role of pharmacoeconomics in current Indian healthcare system. J Res Pharm Pract.

